# The BraveNet prospective observational study on integrative medicine treatment approaches for pain

**DOI:** 10.1186/1472-6882-13-146

**Published:** 2013-06-24

**Authors:** Donald I Abrams, Rowena Dolor, Rhonda Roberts, Constance Pechura, Jeffery Dusek, Sandi Amoils, Steven Amoils, Kevin Barrows, Joel S Edman, Joyce Frye, Erminia Guarneri, Ben Kligler, Daniel Monti, Myles Spar, Ruth Q Wolever

**Affiliations:** 1 UCSF Osher Center for Integrative Medicine, University of California San Francisco, 1545 Divisadero Street, 4th Floor, San Francisco, CA 94115, USA; 2 Duke Clinical Research Institute, Duke University Health System, Durham, NC, USA; 3 Bravewell Collaborative, Minneapolis, MN, USA; 4 Penny George Institute for Health and Healing, Allina Health, Minneapolis, MN, USA; 5 Alliance Institute for Integrative Medicine, Cincinnati, OH, USA; 6 Myrna Brind Center of Integrative Medicine, Thomas Jefferson University, Philadelphia, PA, USA; 7 University of Maryland School of Medicine, Baltimore, MD, USA; 8 Scripps Center for Integrative Medicine, La Jolla, CA, USA; 9 Continuum Center for Health and Healing, Beth Israel Medical Center, New York, NY, USA; 10 Venice Family Clinic, Venice, CA, USA; 11 Duke Integrative Medicine, Duke University Health System, Durham, NC, USA

**Keywords:** Integrative medicine, Chronic pain, Patient-reported outcomes, Complementary therapies, Musculoskeletal disorders

## Abstract

**Background:**

Chronic pain affects nearly 116 million American adults at an estimated cost of up to $635 billion annually and is the No. 1 condition for which patients seek care at integrative medicine clinics. In our Study on Integrative Medicine Treatment Approaches for Pain (SIMTAP), we observed the impact of an integrative approach on chronic pain and a number of other related patient-reported outcome measures.

**Methods:**

Our prospective, non-randomized, open-label observational evaluation was conducted over six months, at nine clinical sites. Participants received a non-standardized, personalized, multimodal approach to chronic pain. Validated instruments for pain (severity and interference levels), quality of life, mood, stress, sleep, fatigue, sense of control, overall well-being, and work productivity were completed at baseline and at six, 12, and 24 weeks. Blood was collected at baseline and week 12 for analysis of high-sensitivity C-reactive protein and 25-hydroxyvitamin D levels. Repeated-measures analysis was performed on data to assess change from baseline at 24 weeks.

**Results:**

Of 409 participants initially enrolled, 252 completed all follow-up visits during the 6 month evaluation. Participants were predominantly white (81%) and female (73%), with a mean age of 49.1 years (15.44) and an average of 8.0 (9.26) years of chronic pain. At baseline, 52% of patients reported symptoms consistent with depression. At 24 weeks, significantly decreased pain severity (−23%) and interference (−28%) were seen. Significant improvements in mood, stress, quality of life, fatigue, sleep and well-being were also observed. Mean 25-hydroxyvitamin D levels increased from 33.4 (17.05) ng/mL at baseline to 39.6 (16.68) ng/mL at week 12.

**Conclusions:**

Among participants completing an integrative medicine program for chronic pain, significant improvements were seen in pain as well as other relevant patient-reported outcome measures.

**Trial Registration:**

ClinicalTrials.gov, NCT01186341

## Background

A 2011 Institute of Medicine report, *Relieving Pain in America*, estimates that chronic pain affects nearly 116 million American adults, a staggering number that surpasses those affected by heart disease, cancer, and diabetes combined [1]. The report concludes that chronic pain costs between $560 billion and $635 billion annually in medical expenses and lost productivity. Although there have been some therapeutic advances, many patients with chronic pain become resistant to conventional medical treatments or suffer adverse effects from widely used prescription medications with high addictive potential, such as non-steroidal anti-inflammatory agents or opiates. For these reasons, patients with chronic pain frequently seek to integrate complementary therapies, often without the knowledge of their primary care provider [2-4].

Integrative medicine provides patient-centered care and addresses the full range of physical, emotional, mental, social, spiritual, and environmental influences that affect a person’s health [5]. Employing a personalized strategy that considers the patient’s unique conditions, needs, and circumstances, integrative medicine uses the most appropriate interventions from an array of scientific disciplines to heal illness and help people regain and maintain optimum health. Because integrative medicine is a “whole systems” approach that employs multiple modalities in concert as opposed to an isolated complementary therapy, studying outcomes is more challenging than evaluating an isolated pharmaceutical or botanical intervention. Chronic pain is one of the main reasons patients seek care at integrative medicine clinics [6,7].

In an effort to capture data on patient-reported outcomes in integrative medicine, a number of leading integrative medicine clinics have collaborated to form the first practice-based research network in the field: the Bravewell Integrative Medicine Research Network (BraveNet). BraveNet’s initial project was to characterize 4182 patients seeking care at nine clinical sites in a systematic fashion by collecting information at a single time point [6]. This repository of data confirmed the high utilization of integrative medicine by chronic pain patients and informed the present Study on Integrative Medicine Treatment Approaches for Pain (SIMTAP). We report the impact of a six-month integrative approach on chronic pain and a number of other related patient-reported outcome measures.

## Methods

### Design overview

Using a prospective, non-randomized, open-label observational design, we assessed patients at baseline and six-, 12-, and 24-week visits. At baseline, participants completed a demographic questionnaire as well as the instruments utilized to assess outcomes over the duration of the study. Within a week of each projected follow-up data collection visit, participants were asked to complete the battery of questionnaires collected at baseline. Information on integrative interventions, prescription medications, and vitamins and supplements utilized was collected at each study visit. Questions about integrative modalities being utilized were answered at weeks six, 12, and 24 to define the scope of the interventions received.

### Setting and participants

Eligible patients were enrolled at each of the nine BraveNet clinical sites. Participants were clinic patients aged 18 or older who were seeking their initial treatment at the center for chronic pain, defined as persisting for at least the past three months with an average level over the past month of at least 4 of 10 on a numerical rating scale. Participants provided written informed consent before initiation of any study-related procedures. The protocol was approved by the Institutional Review Board at each participating site including Allina Health Institutional Review Board; Beth Israel Medical Center Human Subjects Protection Office Institutional Review Board; Duke University Health Systems Institutional Review Board; The Scripps Clinic Institutional Review Board; Thomas Jefferson University Division of Human Subjects Protection; University of California Los Angeles Institutional Review Board; University of California San Francisco Committee on Human Research; and University of Maryland Institutional Review Board.

### Intervention

In keeping with the integrative medicine philosophy of individualized patient-centered care, no standardized prespecified clinical intervention for chronic pain was prescribed for all study participants. Instead, practitioners at each of the network sites devised treatment plans for participating chronic pain patients. All BraveNet sites include integrative physicians, acupuncturists, mindfulness instructors, and yoga instructors; some also incorporate massage therapists, manual medicine therapists, fitness/movement specialists, dietician/nutritionists, psychologists, healing touch therapists, and other energy practitioners.

### Outcomes and follow-up

The battery of validated outcome measurements collected at each visit included the Brief Pain Inventory (BPI) [8], the 12-Item Short Form Health Survey (SF-12v2) [9], the Center for Epidemiologic Studies Depression Scale (CES-D) [10], the Perceived Stress Scale-4 (PSS-4) [11], the Arizona Integrative Outcomes Scale (AIOS) [12], and the Work Productivity and Activity Impairment (WPAI) survey [13]. In addition, numerical rating scales were utilized to assess fatigue, restfulness of sleep, and sense of control. Venipuncture for determination of high-sensitivity C-reactive protein (hs-CRP) (a marker of inflammation) and 25-hydroxyvitamin D levels (potentially associated with pain syndromes) was performed at baseline and week 12.

### Statistical analysis

An objective of this network study was to demonstrate that the sites could enroll and follow patients over a 24-week period and collect serial laboratory specimens. For this analysis, we focus on those participants who completed the entire 24-week program. A post hoc sensitivity analysis was performed to determine potential differences between enrolled patients completing all four study visits (“completers”) and those who completed at least the baseline visit but not the entire study (“noncompleters”). Comparisons of baseline characteristics were assessed utilizing *t*-tests for continuous variables and chi-square tests for categorical measures.

Descriptive statistics were summarized using frequencies (percentages) for categorical variables. Means, standard deviations, medians, quartiles, minimums, and maximums were reported for all continuous variables.

#### Pain and quality-of-life scales

Repeated-measures analyses of variance were performed on study completers to detect changes in BPI subscales—Pain Severity and Interference—over time as well as changes in quality-of-life scores. Results are summarized with a point estimate of mean change in the outcome measures between baseline and any specified follow-up visit with 95% confidence intervals. *P* values are reported for overall model summary, indicating any difference between any two given points between baseline and 24 weeks. If the *P* value was significant (<.05), follow-up linear regression analyses were done to verify that the trends were consistent in direction throughout all study visits, indicating constant improvement or decline from visit to visit.

A secondary sensitivity analysis was done to determine the impact of including only study completers for the primary outcome—BPI Pain Severity and Interference scores—instead of using the more conservative method of last observation carried forward. The repeated-measures analysis for the BPI Pain Severity and Interference scores was run on three subgroups of the sample assuming that values at missed visits were carried forward from the last nonmissing study visit: (1) all enrolled patients, (2) patients completing at least one postbaseline study visit, and (3) patients completing all follow-up study visits.

Multivariate logistic regression analysis was performed to look at variables that influenced the pain response. Responders were defined as those with at least a 20% decrease in the BPI interference score over 24 weeks. Variables included in the model were age, gender, ethnicity (Hispanic or non-Hispanic), years with chronic pain, body mass index, and baseline values for the BPI interference score, SF-12 physical component score, SF-12 mental component score, CES-D score, and PSS-4.

#### Lab measures (hs-CRP and Vitamin D)

We used dependent *t*-tests to determine whether the 12-week study visit laboratory measures differed significantly when compared to baseline values. A *P* value of < .05 was considered to indicate statistical significance.

## Results

A total of 409 participants were consented and enrolled at the nine BraveNet sites between June 2009 and November 2010. The number of participants contributing baseline data per site ranged from 22 to 65 (median: 49). Of the participants, 252 completed all study assessments during the 24-week study and are the basis of our primary analysis.

Table 1 provides baseline demographics of the study completers compared with those who did not contribute data at the 24-week visit. Regardless of completion status, the majority of the participants were women, on average nearly 50 years of age and slightly overweight, who reported experiencing pain for about nine years (completers) or seven years (noncompleters). Other than the duration of pain, completers and noncompleters were similar in demographics and baseline scores on all patient-reported outcomes measured. Subsequent results are presented for the completers only.

**Table 1:** **Baseline measures: completers versus noncompleters**^**a**^

**Measure**	**Completers (*N*** **= 252)**	**Noncompleters (*N*** **= 157)**	***P *Value**^**b**^
Age, *y*	48.6 (15.18)	49.8 (15.89)	.44
Female	74.1%	71.1%	.57
BMI (kg/m^2^)	25.8 (5.49)	25.9 (6.42)	.81
Duration of pain, *y*	8.6 (9.34)	6.9 (9.04)	.06
CES-D (0^c^–60)	17.9 (10.49)	18.3 (10.46)	.71
PSS-4 (0^c^–16)	6.7 (3.14)	6.4 (3.17)	.37
SF-12v2 mental (0–100^c^)	43.5 (10.43)	44.3 (11.17)	.47
SF-12v2 physical (0–100^c^)	37.7 (10.24)	36.8 (10.82)	.40
BPI Severity (0^c^–10)	4.6 (1.86)	4.9 (1.86)	.11
BPI Interference (0^c^–10)	4.6 (2.58)	4.7 (2.47)	.67

*BMI* body mass index, *BPI* Brief Pain Inventory, *CES-D* Center for Epidemiologic Studies Depression Scale, *PSS-4* Perceived Stress Scale-4, *SF-12v2* 12-Item Short Form Health Survey.

^a^Values shown are means (SD).

^b^*P* value indicates significant difference between groups if < .05.

^c^Indicates better health.

Fifty-one percent of the participants reported pain in the neck, 49% lumbar spine, 46% shoulder, 37% hip, 36% knee, 32% sacrum, 31% head, 26% leg, 25% buttock, 25% thoracic spine, and 23% foot. Multiple sites could be selected.

**Figure 1:**
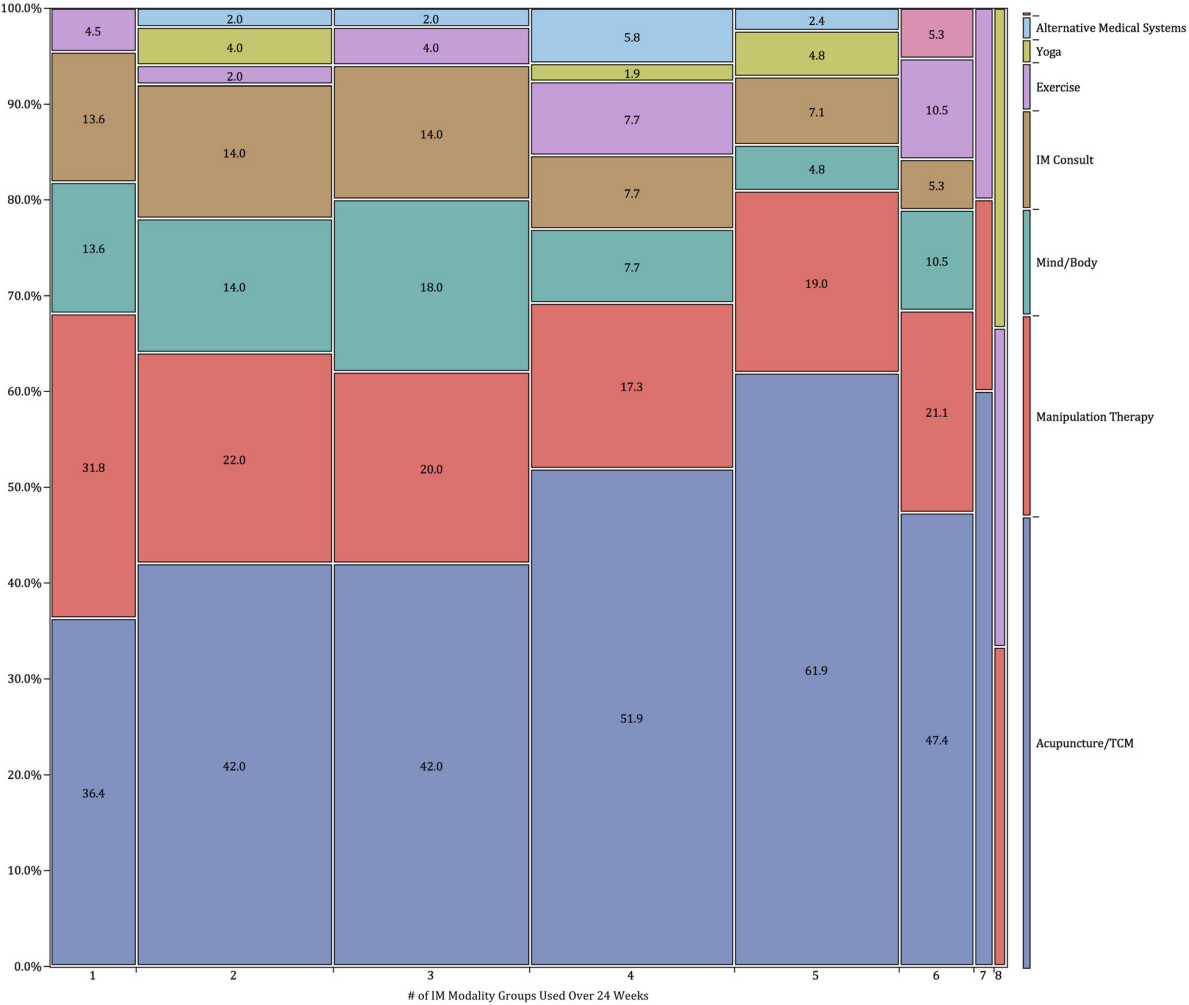
**Mosaic plot of Modalities received by Patients.** The x-axis shows the number of modalities received and the width of the column is proportional to the number of patients in each category – 1 modality (n = 22 patients), 2 modalities (n = 50), 3 modalities (n = 50), 4 modalities (n = 52), 5 modalities (n = 42), 6 modalities (n = 19), 7 modalities (n = 5), all 8 modalities (n = 3). The y-axis shows the proportion of patients who received each modality; the actual percentages are included in each colored box (where feasible).

At week 24, participants were receiving a wide range of modalities in their prescribed integrative suite of therapies (see Table 2). Figure 1 is a mosaic plot of the modalities utilized over the course of the study. The highest number of patients received 4 modalities (N = 55), including acupuncture/Chinese medicine (51.9%), manipulation therapy (17.3%), mind/body techniques (7.7%), integrative medicine consult (7.7%), exercise (7.7%), yoga (1.9%), and alternative medical systems therapy (5.8%). Overall, acupuncture/Chinese medicine, manipulation therapy, and mind/body therapy were the most commonly received modalities.

**Table 2:** **Current integrative medicine pain treatments at 24 weeks for completers (*N*** **= 252)**

**Integrative modality group**^**a**^	**Patients**	**Percentage**
Acupuncture/TCM	115	47.3%
Manipulation Therapy	51	21.0%
Mind/Body	27	11.1%
Integrative Medicine Consult	25	10.3%
Exercise	12	4.9%
Yoga	6	2.5%
Alternative Medical Systems	6	2.5%
Energy Therapy	1	0.4%

^a^ Individual integrative medicine modalities were grouped into eight categories. Acupuncture/TCM: acupuncture, traditional Chinese medicine, tai chi; Manipulation therapy: chiropractic, osteopathy, massage; Mind/Body: biofeedback, deep breathing exercises, hypnosis, meditation, prayer for health, progressive relaxation, psychotherapy; Alternative Medicine Systems: Ayurveda, naturopathy, homeopathy, folk medicine; Energy therapy: healing touch, energy therapy/reiki, Qi gong; Integrative Medicine Consult, Yoga, and Exercise were stand-alone categories.

Current pain medications reported at week 24 included ibuprofen (45%), acetaminophen (32%), naproxen (20%), triptans (18%), hydrocodone (13%), aspirin (11%), and oxycodone (10%). Medication use was unchanged from baseline. Dietary supplements used by more than 10% of participants at week 24 included vitamin D (81%), fish oil (65%), magnesium (32%), green tea (24%), glucosamine (19%), and turmeric (13%). The only changes from baseline in proportion of patients using supplements were seen in vitamin D, magnesium, and glucosamine (55%, 27%, and 26% at baseline, respectively). The mean monthly cost of supplements increased from $47 at baseline to $72 at week 24.

### Pain scores

**Figure 2:**
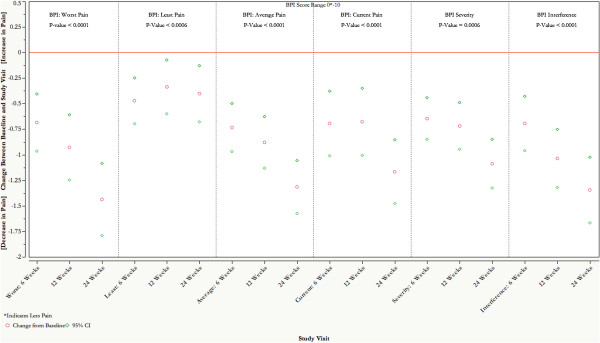
**Repeated-measures analysis of primary outcome: Brief Pain Inventory (BPI) pain scores.** Reported *P* values are overall model results summarizing any potential change across four time points throughout the study. Individual estimated change from baseline scores specify changes in scores from baseline to any designated study visit on a scale from 0 to 10, with 0 indicating least possible pain or interference.

The BPI is composed of four items that assess pain severity and an additional seven questions that assess how the pain interferes with life [8]. The composite score summarizes the patient pain experience over the past 24 hours, with 1–4 rated as mild, 5–6 moderate, and 7–10 severe. The mean baseline severity and interference scores in our cohort were 4.7 (1.86) and 4.7 (2.53), respectively. The overall model results for all three subgroups in both BPI severity and BPI interference scales were statistically significant (*P* < .001). Additionally, mean change scores between baseline and any specific post-baseline study visit for both BPI scales were significantly decreased (*P* < .001). Figure 2 portrays the change in BPI scores over the study duration. Mean severity scores decreased 23% from moderate to mild at 24 weeks. Interference scores dropped 28% to a mean of 3.3 at week 24.

### Depression, stress, and quality of life

Fifty-two percent of completers scored 16 or greater on the CES-D at baseline, consistent with symptoms of depression. As seen in Table 3, by week 24 the mean score had dropped from 17.9 to 13.8 (median score decreased from 16.0 to 11.0); at week 24, only 35% had scores consistent with depressive symptoms. Although both the mental and physical component scores on the SF-12v2 remained below the U.S. norm of 50 at week 24, the mean mental component score rose from 43.5 at baseline to 46.5, and the mean physical component score rose from 37.7 at baseline to 41.5 at week 24 (*P* < .001). Similarly, stress and fatigue decreased over time, and the participants’ sense of control increased (all *P* < .001).

**Table 3:** **Quality-of-life scores by study visit**^**a**^

**Measure**	**Baseline**	**6-week study visit**	**12-week study visit**	**24-week study visit**	**Mean 24-week change from baseline**	***P*value**^**b**^
CES-D (0^c^–60)	17.9 (10.49)	16.1 (10.93)	15.9 (11.83)	13.8 (11.02)	−4.0^d^	<.001
PSS-4 (0^c^–16)	6.7 (3.14)	6.2 (3.21)	5.9 (3.52)	6.1 (3.64)	−1.1^d^	<.001
SF-12v2 mental (0–100^c^)	43.5 (10.44)	45.4 (10.34)	45.6 (10.67)	46.5 (10.97)	3.1^d^	<.001
SF-12v2 physical (0–100^c^)	37.7 (10.24)	39.4 (10.61)	40.7 (10.72)	41.5 (11.56)	3.7^d^	<.001
Quality of sleep (0–10^c^)	4.7 (2.91)	5.0 (2.95)	5.1 (2.82)	5.3 (2.80)	0.7^d^	<.001
Fatigue scale (0^c^–10)	5.8 (2.19)	5.4 (2.32)	5.2 (2.36)	4.9 (2.55)	−0.8^d^	<.001
Sense of control (0–10^c^)	4.8 (2.47)	5.7 (2.28)	5.8 (2.37)	6.1 (2.45)	1.3^d^	<.001

CES-D: Center for Epidemiologic Studies Depression Scale; PSS-4: Perceived Stress Scale-4; SF-12v2: 12-Item Short Form Health Survey.

^a^Values per study visit shown are means (SD); mean 24-week change from baseline values from repeated-measures analysis.

^b^*P* value indicates significant change from baseline and 24-week study visit if < .05; obtained from repeated-measures analysis.

^c^Indicates better health.

^d^Indicates improvement in health.

The AIOS asks participants to summarize their sense of well-being for the entire month by marking a point on a plain line with an “X” somewhere between “Worst you have ever been” and “Best you have ever been.” Participants’ sense of well-being increased significantly from a baseline mean of 41.7 to 55.9 at week 24 (*P* < .001). To put these values in context, rehabilitation center patients scored an average of 33 and healthy controls a 61 on the AIOS [12].

### Work productivity

The WPAI survey assesses the impact of an intervention with respect to daily activity and productivity [13]. It has been previously validated that overall work productivity is significantly related to general health perceptions. The WPAI survey was completed by the 145 participants who reported having employment. The results (see Table 4) demonstrate improvement in all parameters over the 24-week study period.

**Table 4:** **Work productivity and activity impairment survey measures by study visit**^**a**^

**Measure**	**Baseline**	**6-week study visit**	**12-week study visit**	**24-week study visit**	**Mean 24-week change from baseline**	***P*value**^**b**^
Absenteeism (%)	8.5 (17.78)	7.8 (16.36)	4.9 (14.00)	5.1 (14.03)	−3.6^c^	.035
Presenteeism (%)	36.4 (23.54)	32.3 (25.09)	32.2 (26.12)	29.9 (27.99)	−6.9^c^	.002
Work productivity loss (%)	40.8 (25.07)	35.6 (27.67)	33.6 (26.76)	31.0 (28.96)	−10.3^c^	<.001
Activity impairment (%)	51.5 (27.64)	45.7 (26.86)	42.3 (27.67)	40.2 (29.80)	−11.1^c^	<.001

^a^Patients currently employed included in analysis (*N* = 145); values per study visit shown are means (SD); mean 24-week change from baseline values from repeated-measures analysis.

^b^*P* value indicates significant change from baseline and 24-week study visit if < .05; obtained from repeated-measures analysis.

^c^Indicates improvement in health.

### Laboratory studies

Specimens for hs-CRP and 25-hydroxyvitamin D were obtained at baseline and at week 12. Mean hs-CRP decreased from 4.0 (10.21) mg/L at baseline to 2.7 (6.55) mg/L at 12 weeks.

The mean 25-hydroxyvitamin D level at baseline was 33.4 (17.05) ng/mL. At week 12, the mean value had increased to 39.6 (16.68) ng/mL (*P* < .001). Considering >30 ng/mL as the desirable range for 25-hydroxyvitamin D, 52% of the participants were in the desirable range at baseline, and 70% were at week 12 [14].

### Predictors of response

A multivariate logistic regression analysis showed that patients with fewer years of chronic pain (p = 0.03), non-Hispanic populations (p = 0.04), and those with higher (worse) baseline BPI interference scores (p = 0.002) and SF-12 physical component scores (p = 0.008) were more likely to have a clinical meaningful response (i.e. ≥ 20 decrease in the BPI interference score over 24 weeks). Age, gender, BMI, baseline SF-12 mental component score, CES-D score, and PSS-4 score were not significant predictors of response.

## Discussion and conclusion

The initial project of the BraveNet practice-based research network involved collection of a one-time questionnaire from about 500 participants at each of our nine clinical sites [6]. Building on the initial experience of collecting data as a multi-site group, SIMTAP demonstrated our ability to enroll participants and capture outcomes in a prospective longitudinal study, requiring serial follow-up data collection at three time points beyond baseline as well as the ability to collect and process laboratory specimens. The establishment of this functioning practice-based research network in integrative medicine is one of the main accomplishments of this endeavor.

We surpassed the target enrollment of 400, with 409 participants ultimately enrolled. Of these, 88 (21.5%) did not contribute data at the six-week study visit. At 24 weeks, 66% of the enrolled participants were available for follow-up. Chronic pain trials frequently encounter retention issues for a number of reasons, including the nature of the population. It has been suggested that in a typical 12-week, fixed-dose, placebo-controlled trial, a dropout rate of 20%–50% is to be expected [15]. Our participants received no financial incentives for their participation in the 24-week study and, in fact, were responsible for the cost of their integrative treatments. A recent trial on a three-month yoga intervention versus usual care for chronic back pain randomized 156 patients to the yoga arm [16]. Of these, 93 (60%) attended at least three of the first six sessions and at least three others. Acknowledging the need to improve retention, we conclude that within the context of chronic pain intervention trials, the practice-based research network is successful in recruiting and retaining patients who contribute data to assess the impact of the interventions on patient-reported outcomes of interest in a usual care (non-efficacy) setting.

Our study demonstrates that an integrative approach to treating chronic pain had a significant impact on patients’ pain as well as on associated symptoms and quality of life. This success was in the context of long-standing chronic pain, with an average duration in our sample of greater than eight years. Whereas conventional medical interventions, such as pharmaceuticals or surgery, generally focus on one outcome, integrative interventions have the potential to affect multiple aspects of health and well-being [17]. It has been recommended that additional patient-recorded outcome measures are also important to monitor, particularly in studies of chronic pain [18]. The trends in decreased pain, stress, depression, and fatigue, and improvement in physical quality of life and overall well-being, were consistent over the 24-week duration of the trial and suggest the possibility of sustainable effects of the integrative interventions [19]. Particularly notable is the decrease in severity of participants’ depression symptoms, given what is known about the challenges of treating chronic pain and depression. In addition, findings on the WPAI survey suggest that the improvements measured in patient-reported outcomes also translated into greater productivity at work. Predictors of response to an integrative medicine approach included years of chronic pain, ethnicity, and baseline BPI interference and SF-12 physical component scores.

We investigated hs-CRP as a general marker of inflammation rather than as a predictor of cardiac risk [20,21]. The mean hs-CRP value declined one point, suggesting a trend toward decreased inflammation. In a previous study on musculoskeletal pain conducted at our Minneapolis BraveNet site, 93% of the participants were found to have insufficient levels of 25-hydroxyvitamin D (≤20 ng/mL) [22]. To further assess the relationship of hypovitaminosis D to pain, we included vitamin D levels in SIMTAP. Our patients presented with baseline vitamin D levels higher than those seen in the prior study and in the average American adult [21,23]. This likely reflects awareness among both patients and practitioners of the increasing importance of adequate vitamin D levels. The findings that 70% of SIMTAP participants had values greater than 30 ng/mL and none were deficient at week 12 reflect the serious attention paid to maintaining sufficient vitamin D in integrative medicine practices.

### Limitations

One limitation of this study is the loss to follow-up. Our results describe the outcomes of 252 participants at nine sites who completed all study visits during the 24-week integrative medicine intervention for chronic pain. It could be argued that these completers were somehow different from the noncompleters, skewing the final study results, although this notion is not supported by the sensitivity analysis. The bulk of patients lost failed to return for the first study follow-up at week six. This may represent patients who were “shopping” for a new approach for their chronic pain, visited one of the centers, enrolled in SIMTAP, and then chose not to return for follow-up care. The 252 completers can be considered as being treated “on protocol” for analysis in this prospective non-randomized open-label intervention.

A second limitation is the absence of a control group. It could be argued that the significant benefits we observed in SIMTAP participants may have nothing to do with the integrative intervention per se. Because we did not control for natural history and the passage of time, we cannot estimate what proportion of the observed benefits would have occurred separately from the intervention themselves. Without a control group of equal attention, it could be argued that the benefits observed were related to the degree of attention participants received at our centers. Because one of our goals was to assist in the development of our practice-based research network and to demonstrate that we could recruit, follow, and retain participants, as well as collect and process laboratory specimens, we opted not to include randomization or comparison to a control group in our design.

In the spirit of the individualized care that characterizes the integrative medicine approach to the unique individual, we did not mandate a standardized intervention for all SIMTAP participants. Although this makes it more difficult to define the precise treatment that patients received, the network felt that the personalized treatment plan was preferred. Practitioners of traditional Chinese medicine generally devise individualized treatment plans based on each patient’s unique diagnosis rather than follow a standard intervention based on the complaint. In a seminal randomized controlled trial of Chinese herbal medicine for treatment of irritable bowel syndrome, investigators compared a standard and an individualized Chinese herbal formulation to a placebo [24]. Results showed that compared with patients in the placebo group, patients in the two active treatment groups had improvements in bowel symptoms as rated by both the patients and their gastroenterologists at the end of the 16-week intervention. However, at follow-up 14 weeks after completion of the treatment, only the individualized herbal medicine treatment group maintained improvement.

### Future directions

The results of SIMTAP suggest that the tailored, multi-modal approach to treating chronic pain combining conventional and complementary therapies improves quality of life and reduces pain, stress and depressive symptoms. The components of integrative medicine that contribute to improving patients’ physical and emotional health require further research since this study is not powered to compare the effectiveness of different combinations to each other. In addition, a comparison of integrative medicine to usual care would help define the effectiveness of different treatment approaches to chronic pain.

Investigating the therapeutic impact of a “whole systems” approach such as our integrative intervention is a daunting challenge, as funding agencies are accustomed to a more reductionist approach to assessing which individual component of a multimodality intervention is the active one. In addition, the conventional approach would be to deliver a standardized intervention to all study participants. These conventions run counter to the philosophy of integrative medicine, which places the needs of the unique person seeking care first and designs an appropriate personalized intervention based on the individual’s assessment, much akin to practitioners of traditional Eastern medicine. New initiatives, such as the Patient Reported Outcome Measurement Information System and the Patient-Centered Outcomes Research Institute, will hopefully have an impact in advancing research of integrative medicine interventions [25-28].

The 2010 Patient Protection and Affordable Care Act required the Department of Health and Human Services to enlist the Institute of Medicine in examining pain as a public health problem. The recommendations from the institute suggest that among the “steps to improving care, healthcare providers should increasingly aim at tailoring pain care to each person’s experience, and self-management of pain should be promoted” [1]. Although it is often easier and faster to respond to the patient presenting with chronic pain by writing a prescription, increasingly for a substance with addictive potential, an integrative medicine approach may more closely approximate the Institute of Medicine’s blueprint for transforming health care.

## Abbreviations

AIOS: Arizona Integrative Outcomes Scale; BPI: Brief Pain Inventory; BraveNet: Bravewell Integrative Medicine Research Network; CES-D: Center for Epidemiologic Studies Depression Scale; hs-CRP: high-sensitivity C-reactive protein; PSS-4: Perceived Stress Scale-4; SF-12v2: 12-Item Short Form Health Survey; SIMTAP: Study on Integrative Medicine Treatment Approaches for Pain; WPAI: Work Productivity and Activity Impairment.

## Competing interests

CP is a consultant to the Bravewell Collaborative, which funded the creation of BraveNet and this study. The other authors declare that they have no competing interests.

## Authors’ contributions

All authors participated in the conception and design of the study. DIA, RD, RR, RQW carried out the analyses and drafted the manuscript. All authors read and approved the final manuscript.

## Pre-publication history

The pre-publication history for this paper can be accessed here:

http://www.biomedcentral.com/1472-6882/13/146/prepub
